# Case for diagnosis. Unusual involvement of asymptomatic facial papular eruption: eruptive vellus hair cysts^☆^

**DOI:** 10.1016/j.abd.2022.08.011

**Published:** 2023-05-08

**Authors:** Denys Elizabeth Peñaloza Daguer, Alicia Kowalczuk, Mariana Paula Caviedes, Luis Daniel Mazzuoccolo

**Affiliations:** Department of Dermatology, Italian Hospital of Buenos Aires, CABA, Argentina

Dear Editor,

A 44-year-old female patient presented with a medical history of asymptomatic skin lesions covering her face and ears. The lesions had started in puberty with an increasing number since then. She had been treated for acne with topical retinoids, antibiotics, and oral isotretinoin with no improvement. Physical examination showed numerous distinct (1‒3 mm) smooth skin-colored papules concentrated on the cheeks and the ears (Fig. 1 A‒C). There was no family history of similar lesions. A punch biopsy of a papule on the left cheek was performed. The specimen was submitted for histopathological examination (Fig. 2).

**Figure 1:**
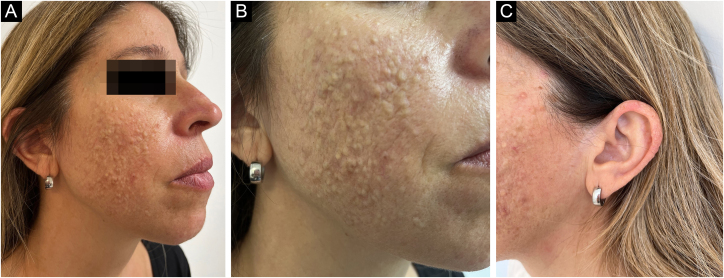
(A‒C) Numerous distinct (1‒3 mm) smooth skin-colored papules concentrated on the cheeks and the ears

**Figure 2:**
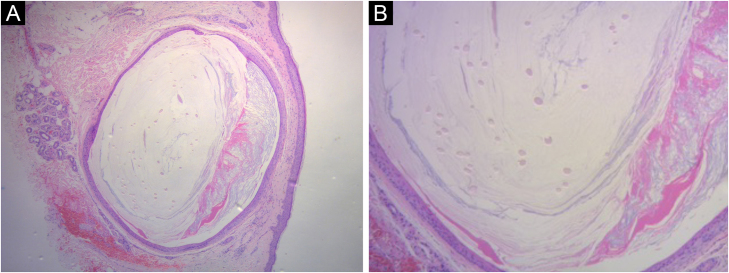
Microscopic Findings. Biopsy specimen from the cheek showing a mid-dermal cyst with abundant lamellated keratin and several vellus hair shafts are present inside the cyst, (A) Hematoxylin & eosin, ×10, (B) Hematoxylin & eosin, ×40.

## What’s your diagnosis?

a. Acneiform eruption

b. Steatocystoma multiplex

c. Epidermal cysts

d. Eruptive vellus hair cysts

## Discussion

After correlating the clinical and histological findings, the diagnosis of eruptive vellus hair cysts (EVHC) with facial involvement was established.

EVHC are a rare benign follicular developmental abnormality of the vellus hair follicles that Esterly and Cols first described in 1977.^1^ They are most commonly seen in children, adolescents, or young adults, affecting different genders and ethnicities equally. They could be sporadic or inherited (autosomal dominant). Furthermore, mutations in the gene that encodes keratin 17 have been described.^1^, ^2^

Clinically, EVHC typically are seen as asymptomatic smooth skin-colored to slightly hyperpigmented follicular papules of 1–4 mm in diameter with a centrally umbilicated surface usually involving the chest, abdomen, and limbs.^3^, ^4^

The facial involvement is uncommon. EVHC has been described as macular, papular, skin-colored, pink, slate hyperpigmented, nevus of Ota-like, and even unilateral. Sites of involvement include the forehead, cheeks, and periorbital areas.^2^ The clinical presentation is often not enough for a definitive diagnosis, which requires a histopathological examination.^2^, ^3^, ^4^

Histologically, they are well-circumscribed cystic lesions in the mid-dermis and/or superficial dermis. The lining epithelium of the cyst wall resembles the infundibular or isthmic portion of the hair follicle and contains two to three layers of stratified squamous epithelium with focal areas of the granular layer. The cyst cavity contains a variable amount of laminated keratin and numerous transversally and obliquely cut vellus hairs. The cyst wall may be in continuity with an atrophied hair follicle or arrector pili muscle. Usually, no sebaceous glands are present within the cyst wall.^3^, ^4^

The most relevant differential diagnosis for this atypical presentation of EVHC is steatocystoma multiplex, which shows a very marked clinical overlap and can be distinguished only by histopathological examination.^4^, ^5^ Other differential diagnoses are acneiform eruptions, milia, and folliculitis.

Although the spontaneous resolution of eruptive vellus hair cysts has been reported, treatment of this condition is often challenging. Therapeutic options include destructive methods such as dermabrasion, excision, and ablative lasers. Topical lactic acid, topical and oral retinoids, and urea creams have also been tried with varying degrees of success.^2^, ^5^

## Financial support

None declared.

## Authors’ contributions

Denys Elizabeth Peñaloza Daguer: Preparation and writing of the manuscript; intellectual participation in propaedeutic and/or therapeutic management of studied case and Study conception and planning.

Alicia Kowalzuck: Intellectual participation in propaedeutic and/or therapeutic management of studied cases.

Mariana Paula Caviedes: Intellectual participation in propaedeutic and/or therapeutic management of studied cases.

Luis Daniel Mazzuoccolo: Critical literature review and approval of the final version of the manuscript.

## Conflicts of interest

None declared.
